# Supplementary material for: The Subjective Experience of Ageism: The Perceived Ageism Questionnaire (PAQ)
Source: Int J Environ Res Public Health. 2022 Jul 19;19(14):8792. doi: 10.3390/ijerph19148792 (PMC9319588; doi:10.3390/ijerph19148792)
Supplement: Supplementary file 1 [file ijerph-19-08792-s001.zip › Supplement S3.pdf]

SUPPLEMENT S3: Path-analysis (Lavaan Code)

```
modelPAQ8 <- "
  #Regressions
  APQ1 ~ a1 * NEG8 + a11 * POS8
  APQ2 ~ a2 * NEG8 + a21 * POS8
  APQ3 ~ a3 * NEG8 + a31 * POS8
  APQ4 ~ a4 * NEG8 + a41 * POS8
  APQ5 ~ a5 * NEG8 + a51 * POS8
  APQ6 ~ a6 * NEG8 + a61 * POS8
  APQ7.1 ~ a7 * NEG8 + a71 * POS8

  #Covariances mediators
  APQ1 ~~ APQ2
  APQ1 ~~ APQ3
  APQ1 ~~ APQ4
  APQ1 ~~ APQ5
  APQ1 ~~ APQ6
  APQ1 ~~ APQ7.1

  APQ2 ~~ APQ3
  APQ2 ~~ APQ4
  APQ2 ~~ APQ5
  APQ2 ~~ APQ6
  APQ2 ~~ APQ7.1

  APQ3 ~~ APQ4
  APQ3 ~~ APQ5
  APQ3 ~~ APQ6
  APQ3 ~~ APQ7.1

  APQ4 ~~ APQ5
```

```
  APQ4 ~~ APQ6
  APQ4 ~~ APQ7.1

  APQ5 ~~ APQ6
  APQ5 ~~ APQ7.1

  APQ6 ~~ APQ7.1

  #Outcome variables
  QOL ~  cp * NEG8 + cp1 * POS8 + c(b1) * APQ1 + c(b2) * APQ2 + c(b3) *
APQ3 + c(b4) * APQ4 + c(b5) * APQ5 + c(b6) * APQ6 + c(b7) * APQ7.1
  MWB ~  cp2 * NEG8 + cp21 * POS8 + c(c1) * APQ1 + c(c2) * APQ2 + c(c3) *
APQ3 + c(c4) * APQ4 + c(c5) * APQ5 + c(c6) * APQ6 + c(c7) * APQ7.1
  DEP ~  cp3 * NEG8 + cp31 * POS8 + c(d1) * APQ1 + c(d2) * APQ2 + c(d3) *
APQ3 + c(d4) * APQ4 + c(d5) * APQ5 + c(d6) * APQ6 + c(d7) * APQ7.1
  ANX ~  cp4 * NEG8 + cp41 * POS8 + c(e1) * APQ1 + c(e2) * APQ2 + c(e3) *
APQ3 + c(e4) * APQ4 + c(e5) * APQ5 + c(e6) * APQ6 + c(e7) * APQ7.1
  LS ~  cp5 * NEG8 + cp51 * POS8 + c(f1) * APQ1 + c(f2) * APQ2 + c(f3) *
APQ3 + c(f4) * APQ4 + c(f5) * APQ5 + c(f6) * APQ6 + c(f7) * APQ7.1
  PSS ~  cp6 * NEG8 + cp61 * POS8 + c(g1) * APQ1 + c(g2) * APQ2 + c(g3) *
APQ3 + c(g4) * APQ4 + c(g5) * APQ5 + c(g6) * APQ6 + c(g7) * APQ7.1

  #Defined effects:
  total_indirect_NEG_APQ_QoL := a1 * b1 + a2 * b2 + a3 * b3 + a4 * b4 + a5
* b5 + a6 * b6 + a7 * b7
  total_indirect_NEG_APQ_MWB := a1 * c1 + a2 * c2 + a3 * c3 + a4 * c4 + a5
* c5 + a6 * c6 + a7 * c7
  total_indirect_NEG_APQ_DEP := a1 * d1 + a2 * d2 + a3 * d3 + a4 * d4 + a5
* d5 + a6 * d6 + a7 * d7
  total_indirect_NEG_APQ_ANX := a1 * e1 + a2 * e2 + a3 * e3 + a4 * e4 + a5
* e5 + a6 * e6 + a7 * e7
  total_indirect_NEG_APQ_LS := a1 * f1 + a2 * f2 + a3 * f3 + a4 * f4 + a5 *
f5 + a6 * f6 + a7 * f7
  total_indirect_NEG_APQ_PSS := a1 * g1 + a2 * g2 + a3 * g3 + a4 * g4 + a5
* g5 + a6 * g6 + a7 * g7
```

```
  total_indirect_POS_APQ_QoL := a11 * b1 + a21 * b2 + a31 * b3 + a41 * b4 +
a51 * b5 + a61 * b6 + a71 * b7

  total_indirect_POS_APQ_MWB := a11 * c1 + a21 * c2 + a31 * c3 + a41 * c4 +
a51 * c5 + a61 * c6 + a71 * c7

  total_indirect_POS_APQ_DEP := a11 * d1 + a21 * d2 + a31 * d3 + a41 * d4 +
a51 * d5 + a61 * d6 + a71 * d7

  total_indirect_POS_APQ_ANX := a11 * e1 + a21 * e2 + a31 * e3 + a41 * e4 +
a51 * e5 + a61 * e6 + a71 * e7

  total_indirect_POS_APQ_LS  := a11 * f1 + a21 * f2 + a31 * f3 + a41 * f4 +
a51 * f5 + a61 * f6 + a71 * f7

  total_indirect_POS_APQ_PSS := a11 * g1 + a21 * g2 + a31 * g3 + a41 * g4 +
a51 * g5 + a61 * g6 + a71 * g7


  "

fit <- lavaan::sem(model = modelPAQ8,data = DATA_PAQ_OUT2, se = "boot",
bootstrap = 5000)

summary(fit, ci = TRUE)

save(fit, file = "fit_Output_path_analysis_revision.Rdata")



> summary(fit, ci = TRUE)


lavaan 0.6-11 ended normally after 214 iterations


  Estimator                                         ML

  Optimization method                           NLMINB

  Number of model parameters                       117


  Number of observations                           500


Model Test User Model:


  Test statistic                                 0.000

  Degrees of freedom                                 0
```

```
Parameter Estimates:

  Standard errors                             Bootstrap
  Number of requested bootstrap draws              5000
  Number of successful bootstrap draws             5000

Regressions:
                   Estimate  Std.Err  z-value  P(>|z|) ci.lower ci.upper
  APQ1 ~
    NEG8      (a1)    0.139    0.040    3.490    0.000    0.059    0.219
    POS8     (a11)    0.030    0.046    0.647    0.518   -0.058    0.122
  APQ2 ~
    NEG8      (a2)    0.304    0.041    7.477    0.000    0.223    0.380
    POS8     (a21)   -0.154    0.043   -3.537    0.000   -0.238   -0.067
  APQ3 ~
    NEG8      (a3)   -0.065    0.029   -2.208    0.027   -0.123   -0.008
    POS8     (a31)    0.169    0.037    4.592    0.000    0.095    0.238
  APQ4 ~
    NEG8      (a4)    0.240    0.043    5.621    0.000    0.156    0.321
    POS8     (a41)   -0.098    0.049   -1.999    0.046   -0.191    0.001
  APQ5 ~
    NEG8      (a5)    0.256    0.037    6.977    0.000    0.182    0.326
    POS8     (a51)   -0.114    0.034   -3.341    0.001   -0.182   -0.047
  APQ6 ~
    NEG8      (a6)   -0.062    0.029   -2.123    0.034   -0.120   -0.005
    POS8     (a61)    0.103    0.035    2.947    0.003    0.032    0.171
  APQ7.1 ~
    NEG8      (a7)    0.118    0.036    3.280    0.001    0.049    0.191
    POS8     (a71)   -0.064    0.037   -1.704    0.088   -0.136    0.010
  QOL ~
    NEG8      (cp)   -0.678    0.135   -5.037    0.000   -0.952   -0.422
```

POS8	(cp1)	0.299	0.153	1.954	0.051	-0.006	0.591
APQ1	(b1)	0.036	0.159	0.228	0.820	-0.274	0.349
APQ2	(b2)	-0.817	0.201	-4.060	0.000	-1.210	-0.421
APQ3	(b3)	0.453	0.239	1.897	0.058	-0.003	0.922
APQ4	(b4)	-0.722	0.188	-3.842	0.000	-1.093	-0.353
APQ5	(b5)	-0.810	0.244	-3.320	0.001	-1.299	-0.327
APQ6	(b6)	0.932	0.239	3.895	0.000	0.457	1.388
APQ7.1	(b7)	-0.025	0.179	-0.140	0.889	-0.372	0.335
MWB ~							
NEG8	(cp2)	-0.200	0.104	-1.926	0.054	-0.402	0.007
POS8	(cp21)	0.479	0.106	4.523	0.000	0.268	0.685
APQ1	(c1)	0.333	0.111	2.995	0.003	0.119	0.558
APQ2	(c2)	-0.645	0.145	-4.451	0.000	-0.938	-0.366
APQ3	(c3)	0.429	0.161	2.661	0.008	0.124	0.749
APQ4	(c4)	-0.118	0.134	-0.877	0.381	-0.381	0.140
APQ5	(c5)	-0.221	0.159	-1.392	0.164	-0.539	0.089
APQ6	(c6)	0.368	0.150	2.445	0.014	0.072	0.657
APQ7.1	(c7)	-0.219	0.133	-1.650	0.099	-0.485	0.040
DEP ~							
NEG8	(cp3)	0.309	0.087	3.572	0.000	0.143	0.479
POS8	(cp31)	-0.313	0.083	-3.778	0.000	-0.473	-0.146
APQ1	(d1)	-0.066	0.087	-0.753	0.452	-0.235	0.105
APQ2	(d2)	0.492	0.108	4.564	0.000	0.287	0.713
APQ3	(d3)	0.081	0.110	0.740	0.459	-0.133	0.295
APQ4	(d4)	0.137	0.102	1.347	0.178	-0.060	0.340
APQ5	(d5)	0.314	0.120	2.605	0.009	0.071	0.548
APQ6	(d6)	-0.190	0.104	-1.834	0.067	-0.390	0.014
APQ7.1	(d7)	-0.057	0.104	-0.548	0.584	-0.262	0.149
ANX ~							
NEG8	(cp4)	0.236	0.068	3.459	0.001	0.105	0.374
POS8	(cp41)	-0.190	0.061	-3.126	0.002	-0.310	-0.071
APQ1	(e1)	-0.009	0.063	-0.140	0.888	-0.135	0.113

```
    APQ2        (e2)    0.322    0.080    4.017    0.000    0.168    0.482
    APQ3        (e3)    0.083    0.076    1.098    0.272   -0.067    0.233
    APQ4        (e4)   -0.050    0.076   -0.658    0.510   -0.199    0.096
    APQ5        (e5)    0.351    0.095    3.701    0.000    0.161    0.540
    APQ6        (e6)   -0.110    0.078   -1.412    0.158   -0.263    0.040
    APQ7.1      (e7)   -0.161    0.080   -2.008    0.045   -0.323   -0.003
  LS ~
    NEG8       (cp5)    0.152    0.052    2.919    0.004    0.053    0.257
    POS8      (cp51)   -0.209    0.057   -3.692    0.000   -0.319   -0.099
    APQ1        (f1)   -0.079    0.059   -1.352    0.176   -0.191    0.038
    APQ2        (f2)    0.106    0.072    1.468    0.142   -0.035    0.246
    APQ3        (f3)   -0.039    0.085   -0.459    0.646   -0.203    0.136
    APQ4        (f4)    0.082    0.068    1.195    0.232   -0.055    0.218
    APQ5        (f5)    0.253    0.095    2.665    0.008    0.066    0.444
    APQ6        (f6)   -0.066    0.089   -0.740    0.459   -0.241    0.111
    APQ7.1      (f7)    0.020    0.073    0.280    0.779   -0.123    0.163
  PSS ~
    NEG8       (cp6)    0.365    0.097    3.749    0.000    0.176    0.560
    POS8      (cp61)   -0.067    0.099   -0.676    0.499   -0.260    0.131
    APQ1        (g1)   -0.000    0.098   -0.005    0.996   -0.195    0.196
    APQ2        (g2)    0.543    0.121    4.477    0.000    0.311    0.785
    APQ3        (g3)   -0.034    0.138   -0.245    0.807   -0.309    0.239
    APQ4        (g4)   -0.066    0.114   -0.576    0.565   -0.287    0.165
    APQ5        (g5)    0.456    0.136    3.359    0.001    0.180    0.720
    APQ6        (g6)   -0.315    0.130   -2.419    0.016   -0.565   -0.062
    APQ7.1      (g7)   -0.191    0.122   -1.570    0.116   -0.427    0.049

Covariances:
                   Estimate  Std.Err  z-value  P(>|z|) ci.lower ci.upper
 .APQ1 ~~
   .APQ2              2.122    0.266    7.968    0.000    1.598    2.649
   .APQ3             -0.568    0.215   -2.635    0.008   -0.995   -0.147
```

.APQ4	2.100	0.275	7.651	0.000	1.557	2.626
.APQ5	1.047	0.208	5.036	0.000	0.627	1.449
.APQ6	-0.397	0.186	-2.135	0.033	-0.765	-0.026
.APQ7.1	0.959	0.202	4.741	0.000	0.555	1.348
.APQ2 ~~						
.APQ3	-0.705	0.183	-3.846	0.000	-1.072	-0.346
.APQ4	2.736	0.245	11.168	0.000	2.240	3.204
.APQ5	2.148	0.193	11.135	0.000	1.763	2.522
.APQ6	-0.548	0.184	-2.979	0.003	-0.904	-0.186
.APQ7.1	0.924	0.210	4.398	0.000	0.508	1.332
.APQ3 ~~						
.APQ4	-0.919	0.195	-4.712	0.000	-1.295	-0.531
.APQ5	-0.724	0.154	-4.703	0.000	-1.022	-0.418
.APQ6	0.559	0.135	4.128	0.000	0.290	0.822
.APQ7.1	-0.631	0.153	-4.120	0.000	-0.923	-0.323
.APQ4 ~~						
.APQ5	1.447	0.207	7.001	0.000	1.038	1.840
.APQ6	-1.092	0.194	-5.618	0.000	-1.465	-0.712
.APQ7.1	1.652	0.240	6.897	0.000	1.173	2.128
.APQ5 ~~						
.APQ6	-0.874	0.162	-5.403	0.000	-1.194	-0.556
.APQ7.1	0.971	0.168	5.779	0.000	0.640	1.298
.APQ6 ~~						
.APQ7.1	-0.981	0.156	-6.279	0.000	-1.276	-0.678
.QOL ~~						
.MWB	16.356	1.875	8.721	0.000	12.372	19.754
.DEP	-10.975	1.355	-8.099	0.000	-13.413	-8.096
.ANX	-5.129	0.958	-5.354	0.000	-6.928	-3.185
.LS	-8.823	0.997	-8.850	0.000	-10.658	-6.665
.PSS	-8.992	1.518	-5.924	0.000	-11.755	-5.790
.MWB ~~						
.DEP	-9.342	1.063	-8.790	0.000	-11.314	-7.140

```
    .ANX              -4.907    0.706   -6.954    0.000   -6.169   -3.442
    .LS               -4.235    0.694   -6.101    0.000   -5.527   -2.773
    .PSS              -9.090    1.092   -8.326    0.000  -11.056   -6.824
  .DEP ~~
    .ANX               5.812    0.664    8.757    0.000    4.406    6.995
    .LS                3.013    0.524    5.745    0.000    1.918    4.000
    .PSS               7.738    0.892    8.676    0.000    5.865    9.321
  .ANX ~~
    .LS                0.921    0.362    2.548    0.011    0.212    1.629
    .PSS               7.202    0.802    8.981    0.000    5.523    8.683
  .LS ~~
    .PSS               1.702    0.559    3.044    0.002    0.557    2.765

Variances:
                   Estimate  Std.Err  z-value  P(>|z|) ci.lower ci.upper
    .APQ1              5.625    0.318   17.689    0.000    4.956    6.211
    .APQ2              5.071    0.284   17.834    0.000    4.490    5.618
    .APQ3              3.145    0.256   12.279    0.000    2.650    3.660
    .APQ4              5.388    0.316   17.055    0.000    4.739    5.979
    .APQ5              3.325    0.226   14.695    0.000    2.879    3.758
    .APQ6              2.775    0.197   14.117    0.000    2.387    3.145
    .APQ7.1            3.574    0.212   16.827    0.000    3.148    3.985
    .QOL              48.291    3.163   15.270    0.000   41.048   53.399
    .MWB              24.858    1.802   13.796    0.000   21.006   27.989
    .DEP              14.866    1.222   12.165    0.000   12.220   17.034
    .ANX               7.975    0.619   12.874    0.000    6.609    9.035
    .LS                7.545    0.508   14.861    0.000    6.394    8.401
    .PSS              21.539    1.587   13.568    0.000   18.095   24.361

Defined Parameters:
                   Estimate  Std.Err  z-value  P(>|z|) ci.lower ci.upper
    tt__NEG_APQ_QL    -0.714    0.103   -6.939    0.000   -0.920   -0.513
```

t__NEG_APQ_MWB	-0.311	0.056	-5.577	0.000	-0.424	-0.205
t__NEG_APQ_DEP	0.253	0.047	5.421	0.000	0.167	0.348
t__NEG_APQ_ANX	0.157	0.031	4.983	0.000	0.096	0.220
tt__NEG_APQ_LS	0.114	0.025	4.540	0.000	0.067	0.166
t__NEG_APQ_PSS	0.265	0.050	5.323	0.000	0.169	0.364
tt__POS_APQ_QL	0.463	0.114	4.048	0.000	0.236	0.687
t__POS_APQ_MWB	0.270	0.059	4.569	0.000	0.160	0.392
t__POS_APQ_DEP	-0.129	0.041	-3.184	0.001	-0.211	-0.052
t__POS_APQ_ANX	-0.072	0.028	-2.614	0.009	-0.129	-0.021
tt__POS_APQ_LS	-0.070	0.023	-2.986	0.003	-0.118	-0.026
t__POS_APQ_PSS	-0.155	0.047	-3.264	0.001	-0.257	-0.067
