# Supplementary material for: The Subjective Experience of Ageism: The Perceived Ageism Questionnaire (PAQ)
Source: Int J Environ Res Public Health. 2022 Jul 19;19(14):8792. doi: 10.3390/ijerph19148792 (PMC9319588; doi:10.3390/ijerph19148792)
Supplement: Supplementary file 1 [file ijerph-19-08792-s001.zip › Supplement S1.pdf]

SUPPLEMENT S1: Original Dutch version of the Perceived Ageism Questionnaire

De volgende vragen gaan over uw ervaringen met de manier waarop anderen u benaderen in het dagelijks leven. Geef telkens aan in hoeverre u onderstaande gevoelens het afgelopen jaar heeft gehad.

	Hoe vaak in het afgelopen jaar heeft u het gevoel gehad dat ..	Nooit	Zelden	Soms	Vaak	Heel
1	.. mensen u vanwege uw leeftijd benaderen alsof u een klein kind bent?	○	○	○	○	○
2	.. er niet naar u geluisterd wordt en/of uw mening/adviezen niet serieus genomen worden vanwege uw leeftijd?	○	○	○	○	○
3	.. mensen uw adviezen en bijdrage aan een gesprek waarderen vanwege uw levenservaring?	○	○	○	○	○
4	.. mensen negatieve vooroordelen of een overdreven stereotype beeldvorming (bijv. zwak, kwetsbaar, saai, langzaam) over u hebben vanwege uw leeftijd?	○	○	○	○	○
5	.. mensen er vanwege uw leeftijd van uitgaan dat u wijs en verstandig bent?	○	○	○	○	○
6	.. mensen u onterecht behandelden alsof u minder aankunt (geestelijk of fysiek) vanwege uw leeftijd?	○	○	○	○	○
7	.. mensen onterecht aannamen dat u niet goed kunt horen vanwege uw leeftijd?	○	○	○	○	○
8	.. mensen vanwege uw leeftijd geringschattend denken (met weinig of geen waarde) over uw bijdrage aan de samenleving?	○	○	○	○	○
9	.. mensen u juist vanwege uw leeftijd als betekenisvol onderdeel van de samenleving zien (bijv. oppassen op (klein)kinderen of vrijwilligersactiviteiten)?	○	○	○	○	○

Note. Rows are color-coded to distinguish items pertaining to the negative (red) and positive (blue) subscale of the Perceived Ageism Questionnaire (PAQ). Item 7 was dropped from the final scale (PAQ-8) and is therefore colored grey.
