# Supplementary material for: The Subjective Experience of Ageism: The Perceived Ageism Questionnaire (PAQ)
Source: Int J Environ Res Public Health. 2022 Jul 19;19(14):8792. doi: 10.3390/ijerph19148792 (PMC9319588; doi:10.3390/ijerph19148792)
Supplement: Supplementary file 1 [file ijerph-19-08792-s001.zip › Supplement S2.pdf]

SUPPLEMENT S2: Supplementary Results

Table S1. Inter-item correlations for the PAQ items of Analysis 1 (vertical and Analysis 2 (Horizontal).

2<br>1	**1**	**2**	**3**	**4**	**5**	**6**	**7**	**8**	**9**
**1**		0.47	0.009	0.38	−0.03	0.38		0.44	0.04
**2**	**0.51**		0.11	0.49	0.09	0.44		0.42	0.10
**3**	−0.11	−0.12		0.002	**0.54**	−0.02		−0.004	0.40
**4**	0.44	0.42	−0.09		0.11	0.46		0.49	0.008
**5**	−0.06	−0.08	**0.60**	−0.01		0.004		0.06	0.44
**6**	0.44	0.43	−0.07	0.52	0.03			0.44	0.006
**7**	0.26	0.27	0.03	0.29	0.03	0.35			
**8**	0.41	0.42	−0.09	0.46	−0.07	**0.52**	0.44		0.01
**9**	−0.04	0.005	0.48	−0.02	0.47	0.006	0.12	−0.01	

Note. Item 7 was removed from the matrix in Analysis 2, as a PAQ-8 was found to be reliable in Analysis 1.

Table S2. *Standardized loadings for two-factor confirmatory model of the 8-item perceived ageism questionnaire (PAQ) – Analysis 2*

	**Negative (NEG)**	**Positive (POS)**
**Item**	Standardized loadings	Standardized loadings
1	0.61	
2	0.69	
3		0.70
4	0.70	
5		0.77
6	0.63	
7		
8	0.67	
9		0.57

Note. NEG: perceived negative ageism subscale, POS: perceived positive ageism subscale.

Table S3. *Descriptives of perceived negative (NEG) and positive (POS) ageism per age group – Analysis 2*

		NEG			POS		
		M	SD	min - max	M	SD	min - max
**Young-old (<= 67)**	n = 263	6.09	2.37	5 – 16	9.32	2.36	3 – 15
**Old-old (> 67)**	n = 237	7.43	2.66	5 – 19	9.87	2.19	3 – 15

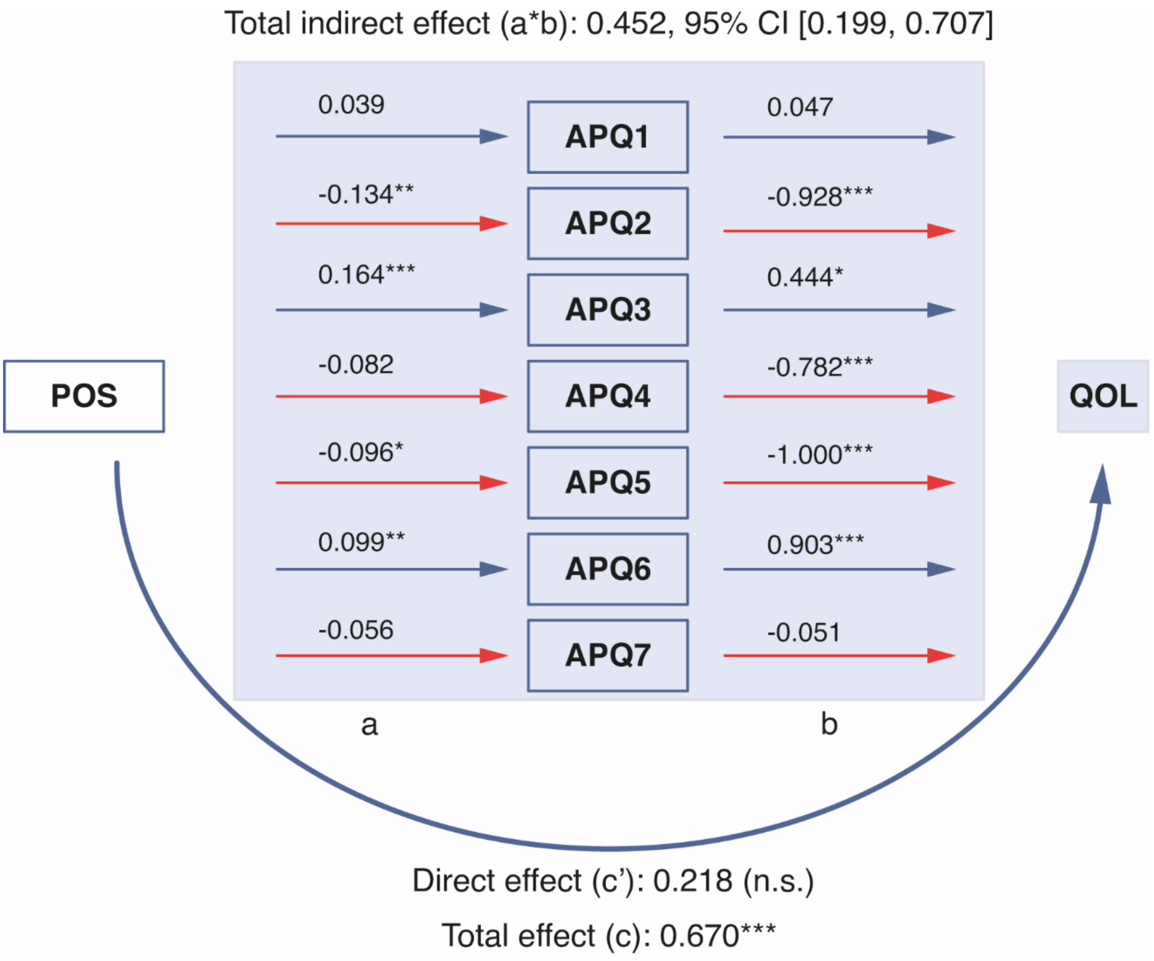

Figure S1. *Mediation Diagram for the Relationship Between Positive (POS) Perceived Ageism and Quality of Life (QOL) as Mediated by Self-perceptions of Aging (APQ)*

Note. Values represent unstandardized regression coefficients. *$p < .05$, **$p < .01$, ** < .001*

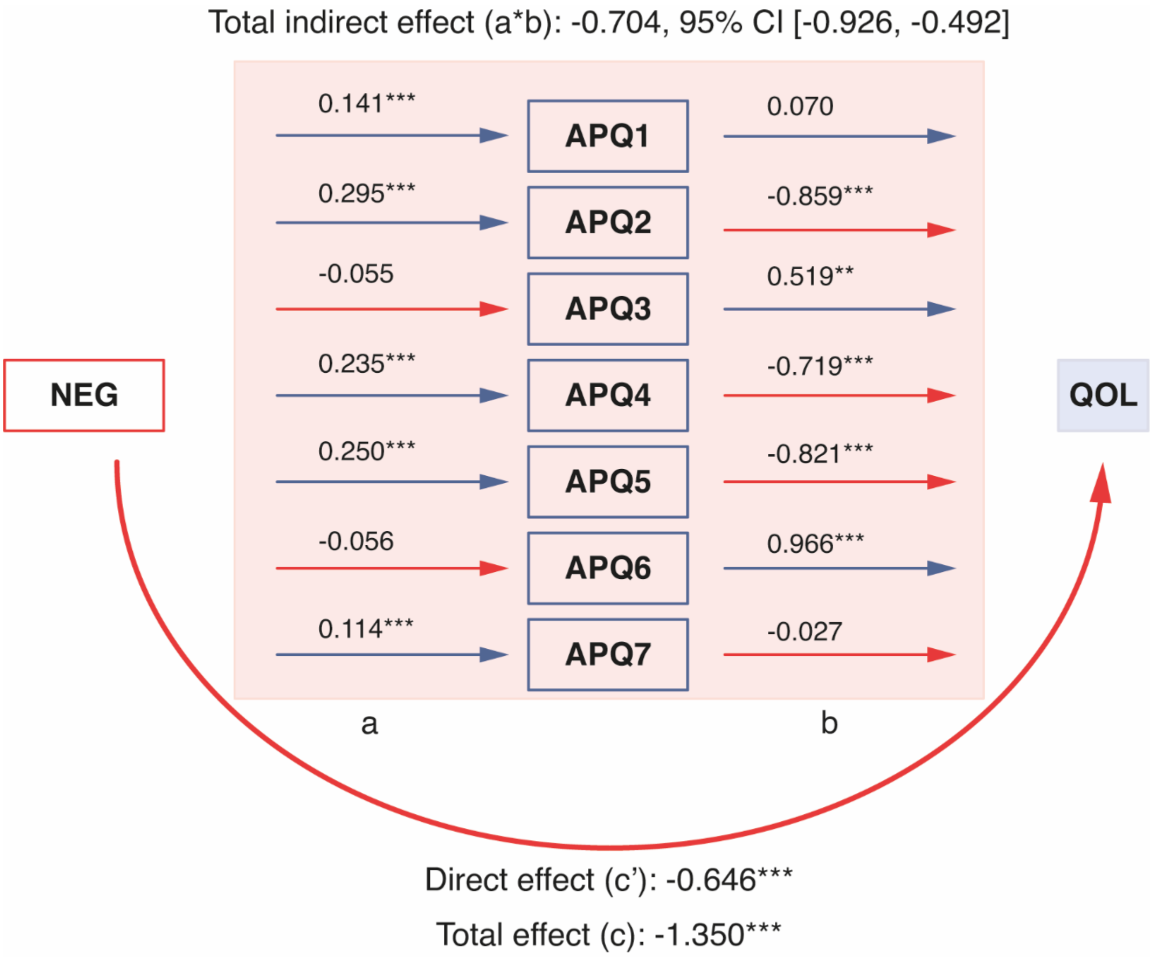

Figure S2. *Mediation Diagram for the Relationship Between Negative (NEG) Perceived Ageism and Quality of Life (QOL) as Mediated by Self-perceptions of Aging (APQ)*

Note. Values represent unstandardized regression coefficients. *$p < .05$, **$p < .01$, ** $< .001$

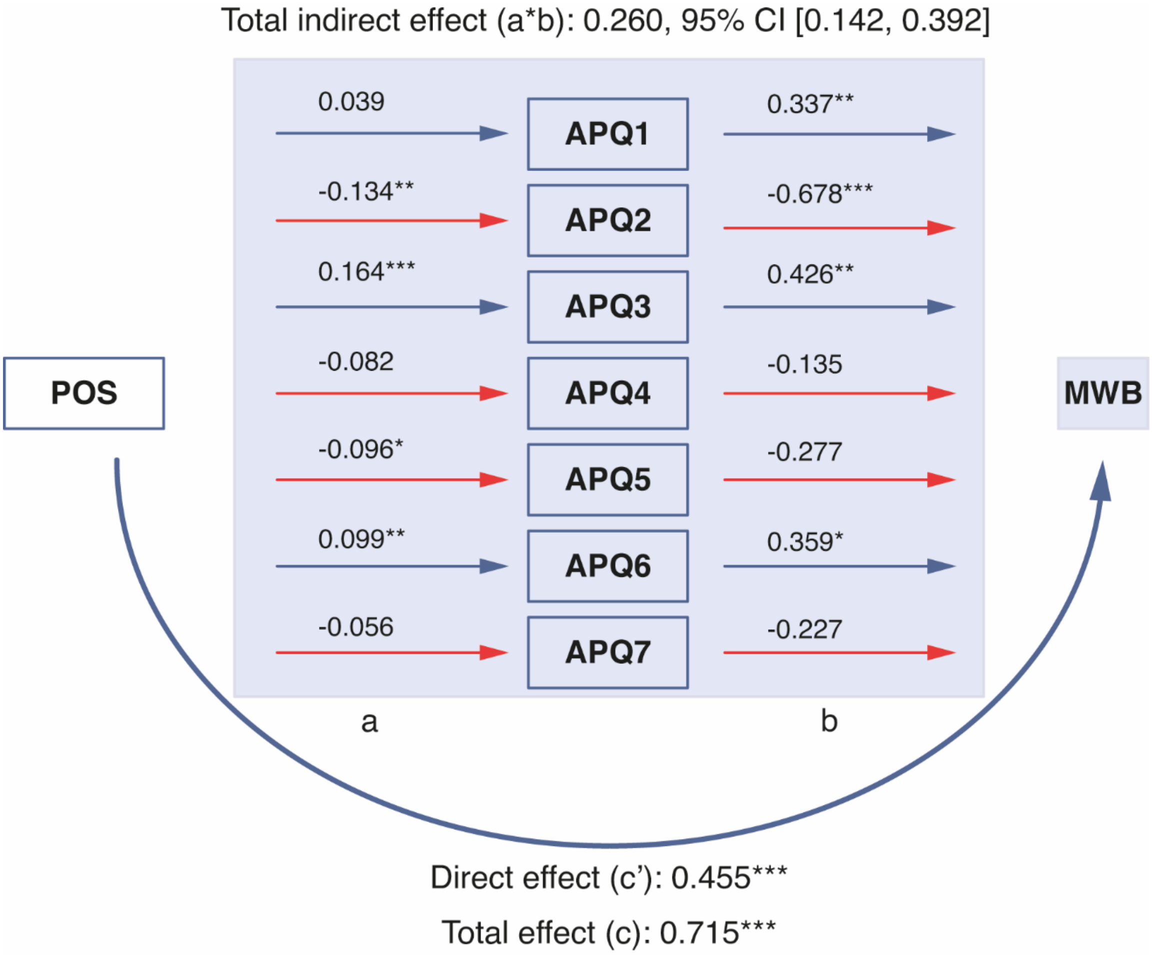

Figure S3. *Mediation Diagram for the Relationship Between Positive (POS) Perceived Ageism and Mental Well-being (MWB) as Mediated by Self-perceptions of Aging (APQ)*

Note. Values represent unstandardized regression coefficients. $*p < .05$, $**p < .01$, $** < .001$

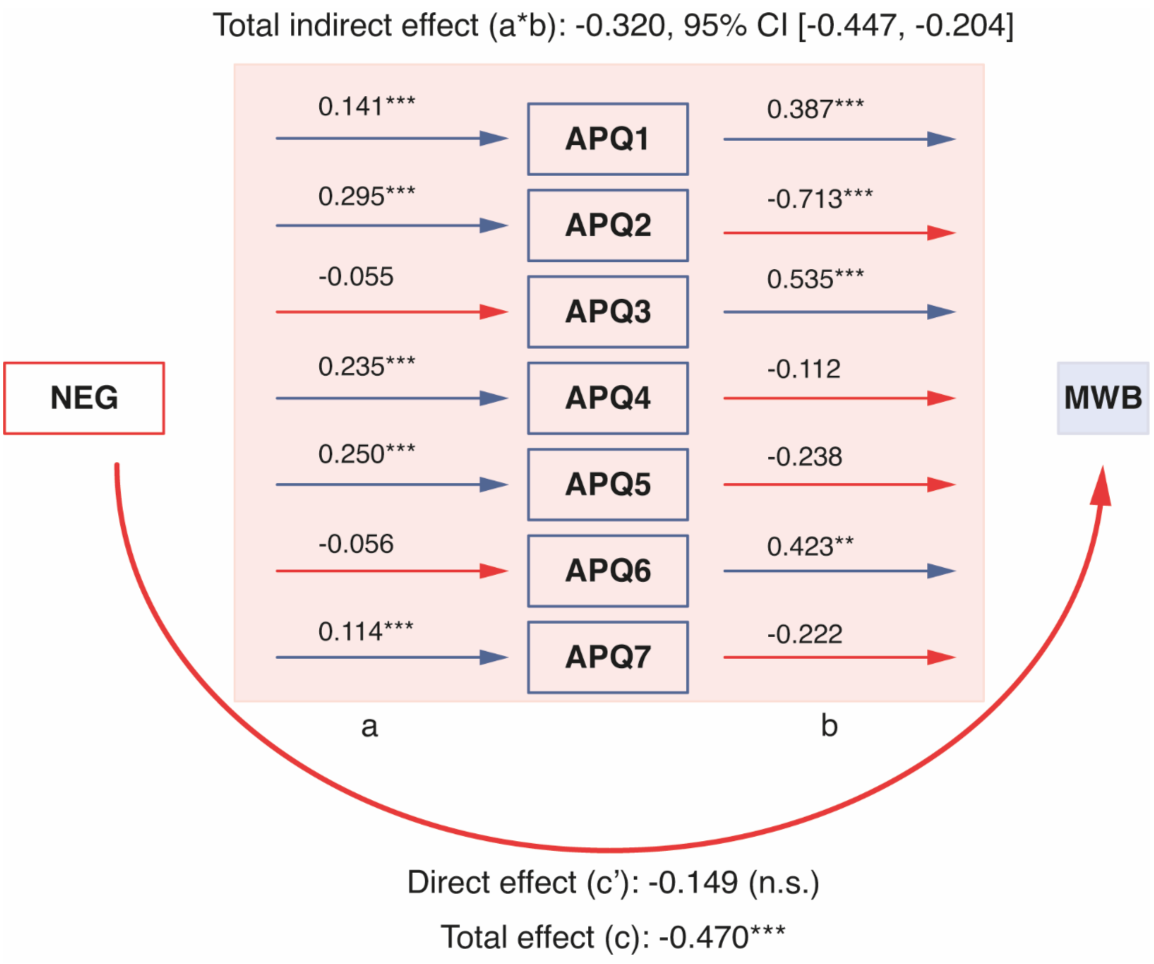

Figure S4. *Mediation Diagram for the Relationship Between Negative (NEG) Perceived Ageism and Mental Well-being (MWB) as Mediated by Self-perceptions of Aging (APQ)*

Note. Values represent unstandardized regression coefficients. *$p < .05$, **$p < .01$, ** < .001

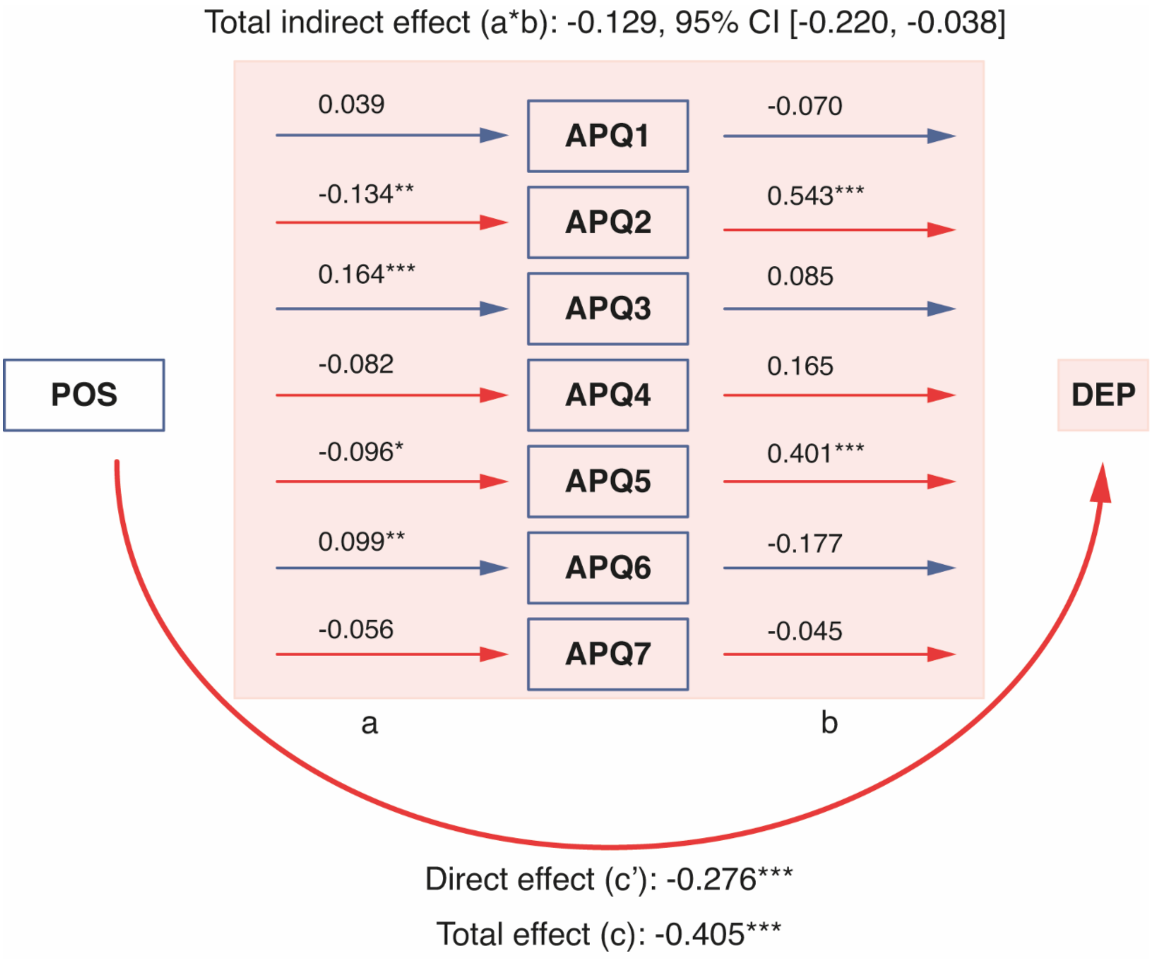

Figure S5. *Mediation Diagram for the Relationship Between Positive (POS) Perceived Ageism and Depression (DEP) as Mediated by Self-perceptions of Aging (APQ)*

Note. Values represent unstandardized regression coefficients. *p < .05, **p < .01, ** < .001*

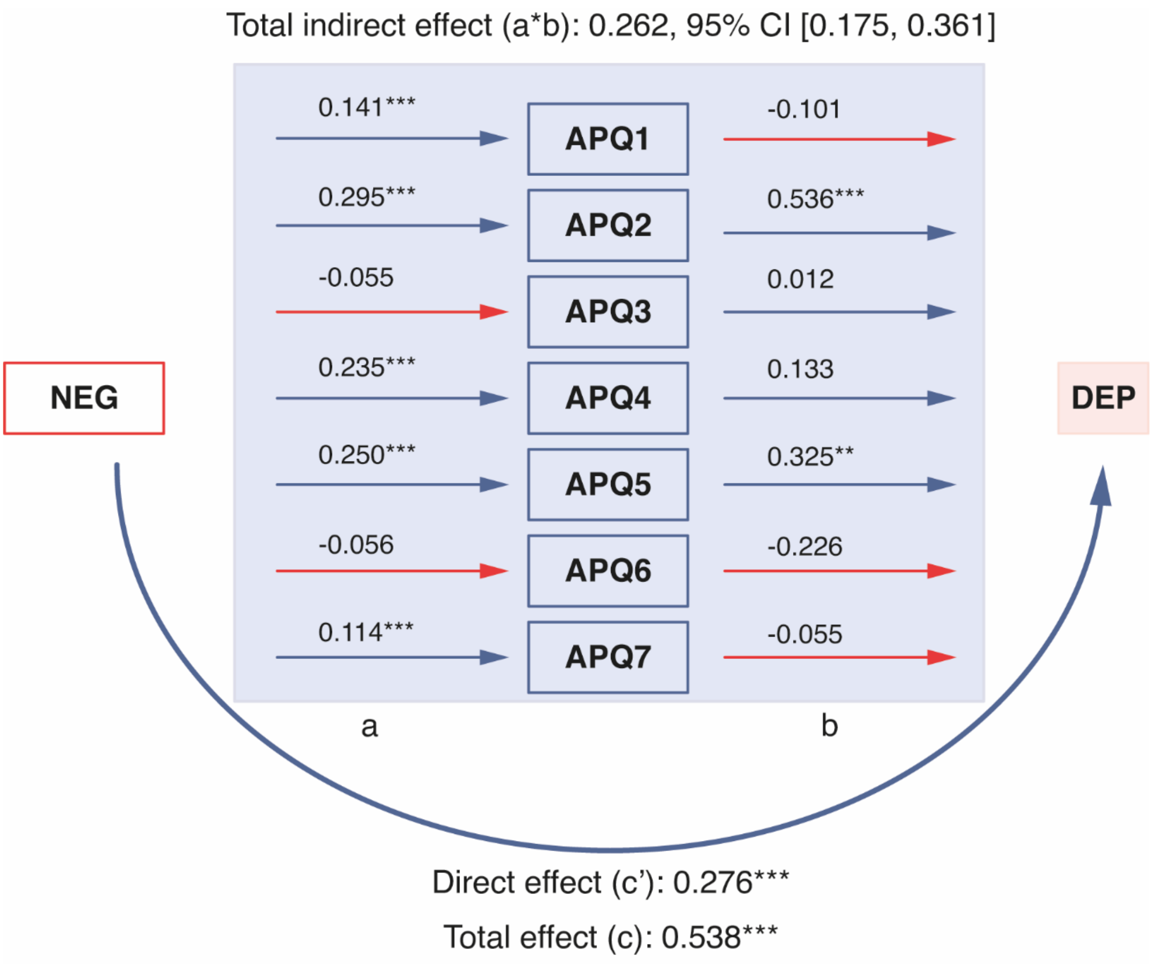

Figure S6. *Mediation Diagram for the Relationship Between Negative (NEG) Perceived Ageism and Depression (DEP) as Mediated by Self-perceptions of Aging (APQ)*

Note. Values represent unstandardized regression coefficients. *$p < .05$, **$p < .01$, ** < .001*

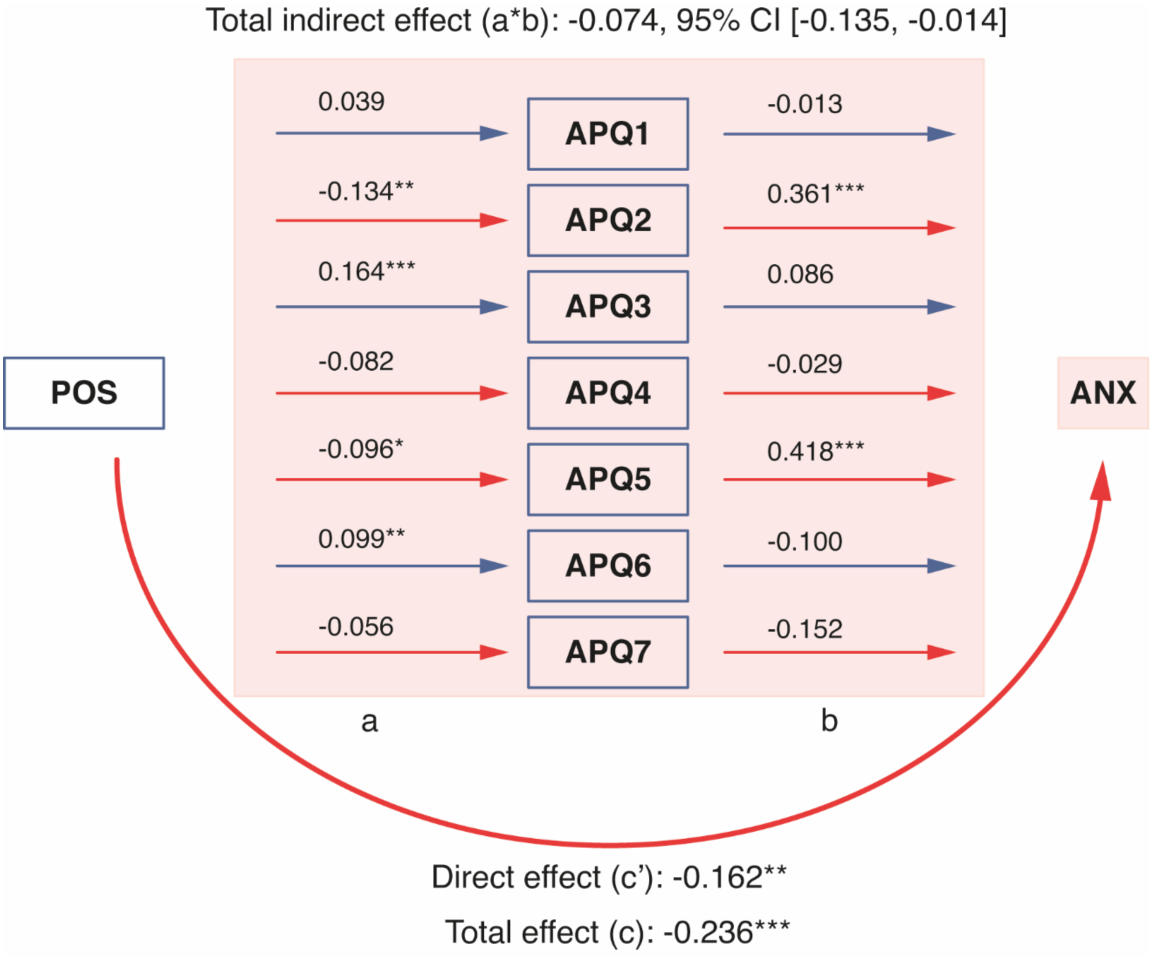

Figure S7. *Mediation Diagram for the Relationship Between Positive (POS) Perceived Ageism and Anxiety (ANX) as Mediated by Self-perceptions of Aging (APQ)*

Note. Values represent unstandardized regression coefficients. *$p < .05$, **$p < .01$, ** $< .001$

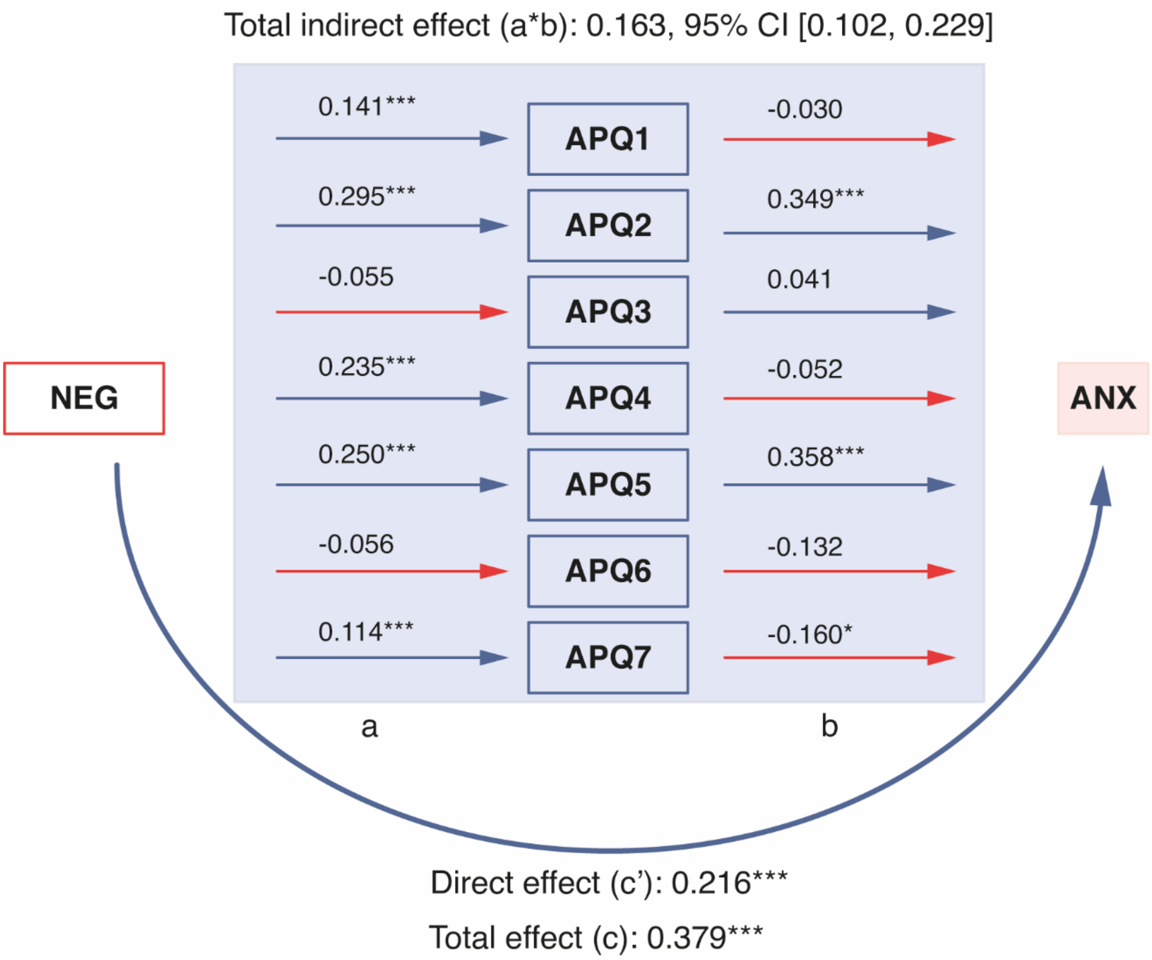

Figure S8. *Mediation Diagram for the Relationship Between Negative (NEG) Perceived Ageism and Anxiety (ANX) as Mediated by Self-perceptions of Aging (APQ)*

Note. Values represent unstandardized regression coefficients. *$p < .05$, **$p < .01$, ** $< .001$

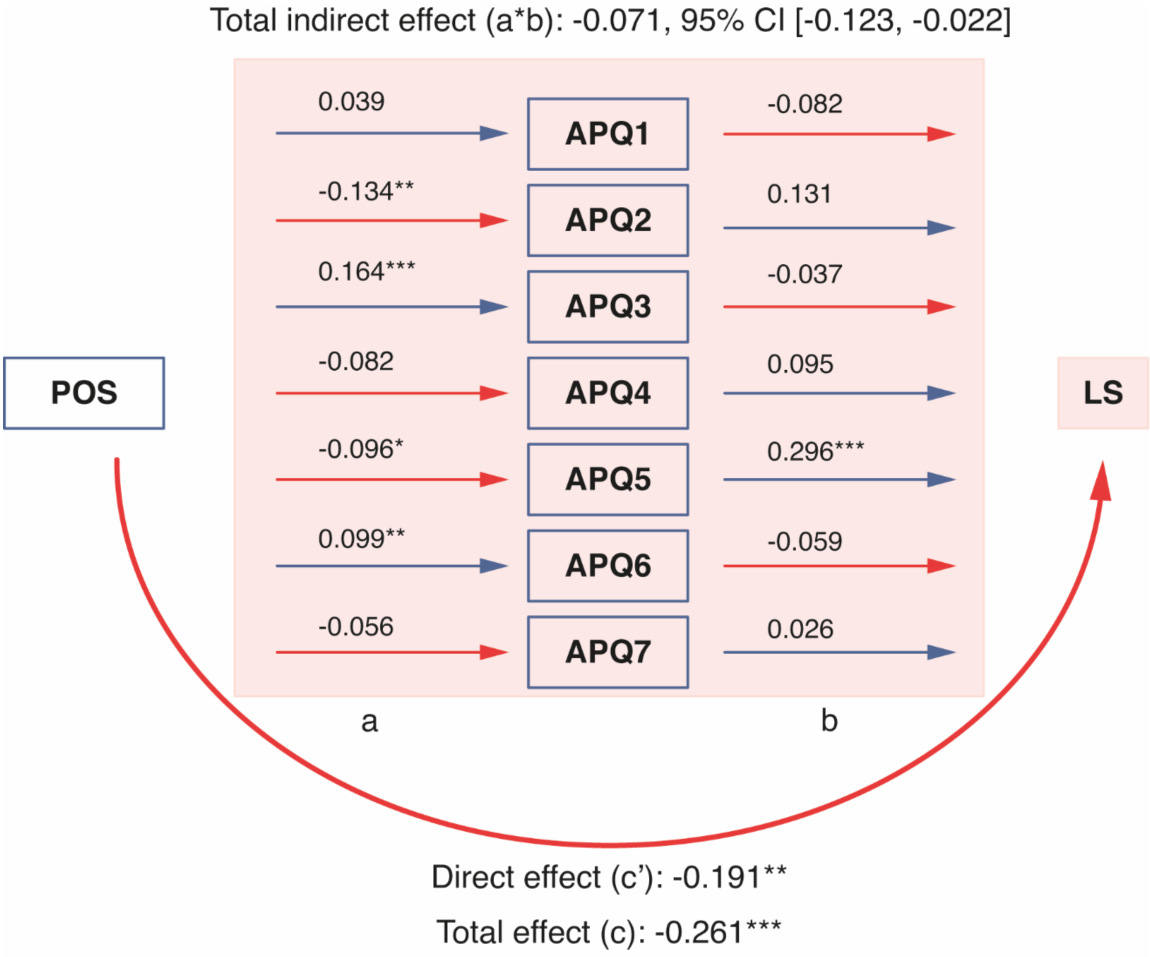

Figure S9. *Mediation Diagram for the Relationship Between Positive (POS) Perceived Ageism and Loneliness (LS) as Mediated by Self-perceptions of Aging (APQ)*

Note. Values represent unstandardized regression coefficients. *$p < .05$, **$p < .01$, ** $< .001$

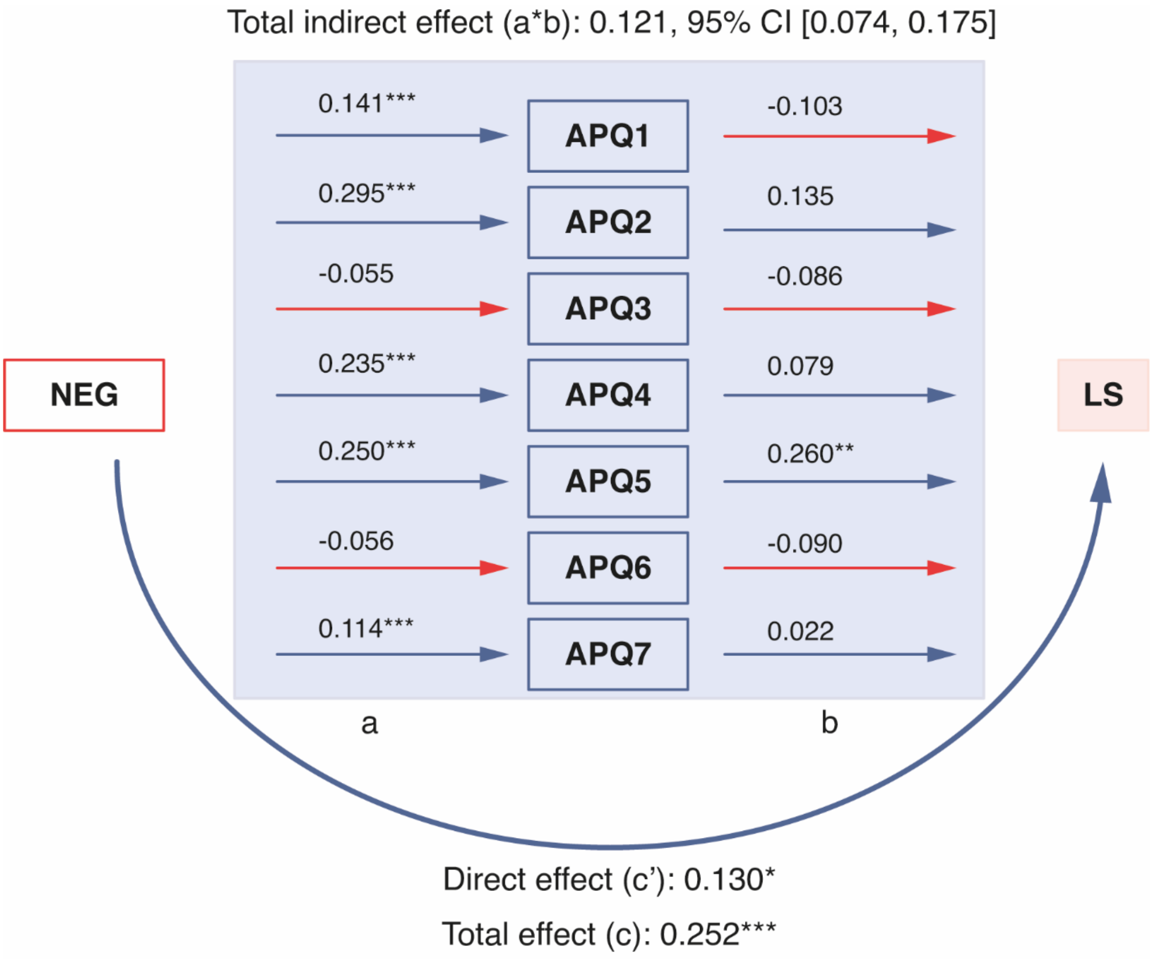

Figure S10. *Mediation Diagram for the Relationship Between Negative (NEG) Perceived Ageism and Loneliness (LS) as Mediated by Self-perceptions of Aging (APQ)*

Note. Values represent unstandardized regression coefficients. *$p < .05$, **$p < .01$, ** < .001

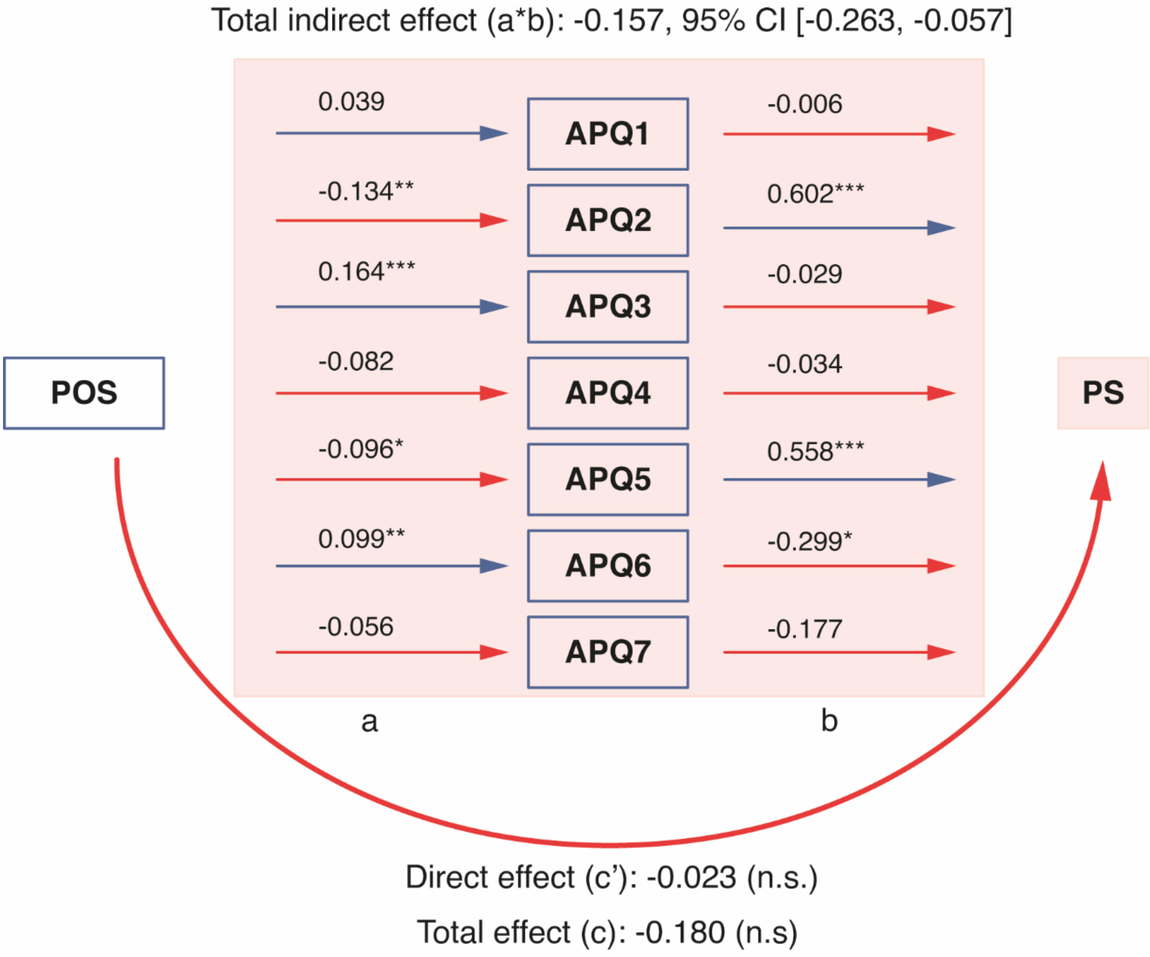

Figure S11. *Mediation Diagram for the Relationship Between Positive (POS) Perceived Ageism and Perceived Stress (PS) as Mediated by Self-perceptions of Aging (APQ)*

Note. Values represent unstandardized regression coefficients. *$p < .05$, **$p < .01$, ** < .001*

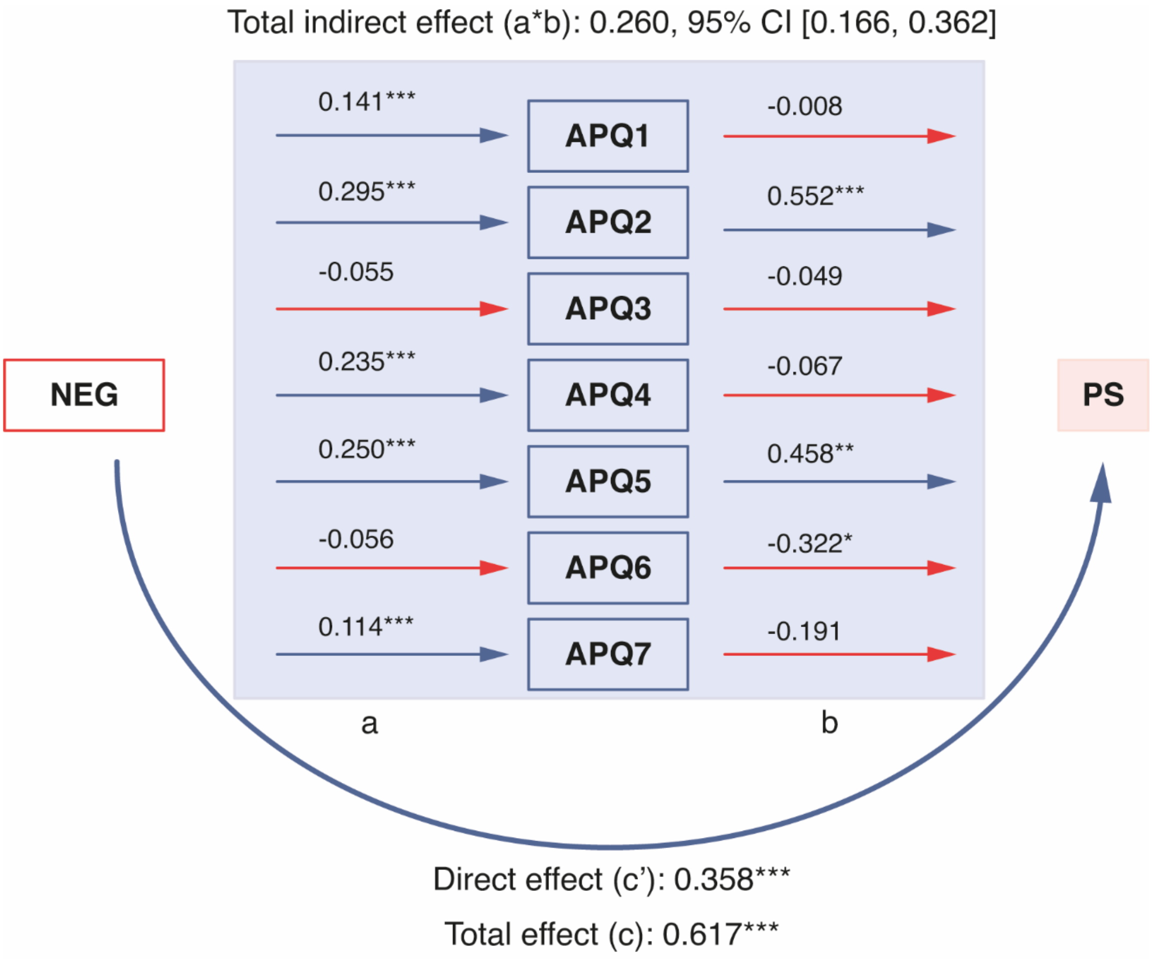

Figure S12. *Mediation Diagram for the Relationship Between Negative (NEG) Perceived Ageism and Perceived Stress (PS) as Mediated by Self-perceptions of Aging (APQ)*

Note. Values represent unstandardized regression coefficients. *$p < .05$, **$p < .01$, ** $< .001$

Table S4. Direct and indirect effects, via self-perceptions of aging, of the mediation models.

		**NEG** (r)			**POS** (r)		
		B	SE	95% CI	B	SE	95% CI
Depression	Direct	0.309***	0.087	[0.14, 0.48]	−0.313***	0.083	[−0.47, −0.15]
	Indirect	0.253	0.047	[0.17, 0.35]	−0.129	0.041	[−0.21, −0.05]
Anxiety	Direct	0.236***	0.068	[0.11, 0.37]	−0.190**	0.061	[−0.31, −0.07]
	Indirect	0.157	0.031	[0.10, 0.22]	−0.072	0.028	[−0.13, −0.02]
Loneliness	Direct	0.152**	0.052	[0.05, 0.26]	−0.209***	0.057	[−0.32, −0.10]
	Indirect	0.114	0.025	[0.07, 0.17]	−0.070	0.023	[−0.12, −0.03]
Perceived stress	Direct	0.365***	0.097	[0.18, 0.56]	−0.067	0.099	[−0.26, 0.13]
	Indirect	0.265	0.050	[0.17, 0.36]	−0.155	0.047	[−0.26, −0.07]
Quality of Life	Direct	−0.678***	0.135	[−0.95, −0.42]	0.299	0.153	[−0.01, 0.59]
	Indirect	−0.714	0.103	[−0.92, −0.51]	0.463	0.114	[0.24, 0.69]
Mental well-being	Direct	−0.200	0.104	[−0.40, 0.00]	0.479***	0.106	[0.27, 0.69]
	Indirect	−0.311	0.056	[−0.42, −0.21]	0.270	0.059	[0.16, 0.39]

Note. NEG: perceived negative ageism subscale, POS: perceived positive ageism subscale. B represents unstandardized regression weights. Square brackets are used to enclose the lower and upper limits of a confidence interval (CI). Outcomes are derived from a path-analysis, including all paths of interest, estimated with Maximum Likelihood. **p* < .05, ***p* < .01, ****p* < .001
